# MGMT promoter methylation in Peruvian patients with glioblastoma

**DOI:** 10.3332/ecancer.2018.812

**Published:** 2018-02-14

**Authors:** Carolina Belmar-Lopez, Carlos A Castaneda, Miluska Castillo, Pamela García-Corrochano, Enrique Orrego, Barbara Meléndez, Sandro Casavilca, Claudio Flores, Enrique Orrego

**Affiliations:** 1 Department of Research, Instituto Nacional de Enfermedades Neoplasicas. Av Angamos Este 2520, Surquillo, Lima 15038, Peru; 2 Faculty of Medicine, Universidad Peruana San Juan Bautista. Av Jose Antonio Lavalle s/n, Chorrillos, Lima 15067, Peru; 3 Department of Neurosurgery, Instituto Nacional de Enfermedades Neoplasicas, Lima 15038, Peru; 4 Molecular Pathology Research Unit, Department of Pathology, Virgen de la Salud Hospital. Av De Barber s/n, Toledo 45005, Spain; 5 Department of Pathology, Instituto Nacional de Enfermedades Neoplasicas. Av Angamos Este 2520, Surquillo, Lima 15038, Peru; 6 Department of Research, Oncosalud. Av Guardia Civil 571, San Borja, Lima 15036, Peru

**Keywords:** MGMT, methylation, glioblastoma, Latin American

## Abstract

**Purpose:**

O^6^-methylguanine–DNA methyltransferase (*MGMT*) promoter methylation predicts the outcome and response to alkylating chemotherapy in glioblastoma. The aim of this study is to evaluate the prevalence of *MGMT* methylation in Peruvian glioblastoma cases.

**Patients and methods:**

We evaluated retrospectively 50 cases of resected glioblastoma during the period 2008–2013 at Instituto Nacional de Enfermedades Neoplasicas in Peru. Samples consisted of paraffin embedded and frozen tumour tissue. *MGMT*-promoter methylation status and the expression level of *MGMT* gene were evaluated by methylation-specific PCR and real-time PCR, respectively.

**Results:**

Unmethylated, methylated and partially methylated statuses were found in 54%, 20% and 26% of paraffin-embedded samples, respectively. Methylation status was confirmed in the Virgen de la Salud Hospital and frozen samples. There was an association between the status of *MGMT*-promoter methylation and the level of gene expression (*p* = 0.001). Methylation was associated with increased progression-free survival (*p* = 0.002) and overall survival (OS) (*p* < 0.001).

**Conclusion:**

*MGMT*-promoter methylation frequency in Peruvian glioblastoma is similar to that reported in other populations and the detection test has been standardised.

## Background

Glioblastoma is the most common primary malignant brain tumour in adults and is invariably associated with an unfavourable prognostic. Patient survival rarely exceeds 12 months [1, 2]. Recent studies showed that the combined use of radiation and alkylating agents, such as temozolomide, increases the survival of patients with glioblastoma [3].

O^6^-methylguanine-DNA methyltransferase (MGMT) is a DNA repair enzyme that rapidly reverses alkylation of the O^6^ position of guanine and neutralises the cytotoxic effect of the mentioned alkylating agents [4]. The *MGMT* promoter contains a CpG island rich in Cytosine-Guanine (CG) dinucleotides or CpG sites and is positioned in the 5’ region of the same *MGMT* gene, located on the long arm of chromosome 10. Methylation of the CpG sites produces binding to specific proteins, and this complex causes altered chromatin structures and loss of transcription. Epigenetic silencing of the *MGMT* gene can be detected in around half of the glioblastoma cases and is associated with increased survival in patients receiving alkylating agents [5–11].

Stupp *et al*. evaluated the methylation status of *MGMT*-promoter in 206 glioblastomas through the specific PCR methylation test and found promoter methylation in 45%. It was associated with both longer overall survival (OS) and progression-free survival (PFS) in the treatment arm of concurrent radiation along with temozolomide (*p* < 0.007) [3, 6, 7].

The processes of methylation and other epigenetic changes are generated by the influence of environmental factors (e.g., food and pollution) and phenotypic characteristics (e.g., obesity) that vary according to the socio-cultural characteristics of populations [12].

The objective of the present study was the epidemiological evaluation of the methylation of the *MGMT* promoter in Peruvian patients with glioblastoma and the standardisation of the PCR technique in paraffin-embedded [formalin-fixed paraffin-embedded (FFPE)] samples from the Instituto Nacional de Enfermedades Neoplasicas (INEN) in Lima, Peru. This information will allow us, for the first time to our knowledge, to be aware of epidemiological information of epigenetic changes that behave as predictive biomarkers of response to treatment in the Peruvian population with implications for Public Health. The standardisation process includes four steps: (*i*) methylation status analysis of paired frozen and FFPE tumour samples (*n* = 7), and subsequent extension to 43 additional FFPE samples; (*ii*) validation test of methylation status in two evaluated FFPE samples at other institutions; (*iii*) *MGMT* gene expression analysis in frozen tumour (*n* = 7) and (*iv*) evaluation of the clinical behaviour of glioblastoma cases with methylated MGMT promoter.

## Materials and methods

### Study population

The study population consisted of 50 newly diagnosed glioblastoma cases resected during the period 2008–2013 at INEN with available FFPE samples. Seven cases had paired frozen tissue (ID: 21, 23, 24, 26, 27 and 40) stored at pathology department of our institute. Two FFPE samples (ID: 4 and 7) were also evaluated in the Molecular Pathology Research Unit of the Molecular Pathology Research Unit, Hospital Virgen de la Salud de Toledo, Spain (HVST).

Clinical and epidemiological information were obtained from the medical records and include: age, sex, initial Karnofsky performance status, tumour location, type of resection, adjuvant treatment, recurrence, recurrence date, follow-up date, and date of death.

### Tumour area selection

Tumour samples were evaluated on haematoxylin–eosin slides for the identification of viable tumour areas (≥ 80%) with exclusion of necrotic, haemorrhagic or acute inflammatory reaction areas. A 0.6 cm cylinder was removed from the selected area and included in a new paraffin block.

### Extraction of nucleic acids

For DNA extraction in frozen tissue, 15–20 mg from each sample was homogenised and purified using the GeneJET Genomic DNA Purification Kit (Thermo Scientific), following the manufacturer’s directions. For DNA and RNA extraction in FFPE samples, six slides (10-*μ*m-thick sections) were processed and purified according to the commercial GeneJET FFPE DNA Purification kit (Thermo Scientific) and PureLink FFPE Total RNA Isolation Kit (Invitrogen, Life Technologies), repectively.

### Quantification of nucleic acids

A fluorometric reading was performed to determine the concentrations of DNA and RNA obtained in the extractions. Measurements were carried out with Qubit (Invitrogen, Life Technologies) whose values are given in ng/μl. For this, a 1:200 working solution was prepared with the dsDNA/HS or RNA/HS fluorophore. For the quantification, 1 *μ*l of DNA or RNA was added to the 199 *μ*l working solution, and the samples were read.

### DNA modification

Through treatment with bisulfite, unmethylated cytosine residues were converted to uracil. Two commercial kits were employed: (*i*) EpiJET Bisulfite Conversion Kit (Thermo Scientific), whereby 500 ng of the DNA was subjected to bisulfite, following the manufacturer’s directions. 120 μl of DNA-modifying reagent was added, and denaturation and subsequent conversion with bisulfite (98°C 10 minutes, 60°C 150 minutes) were performed. The DNA was then purified using the columns of the kit. (*ii*) EpiTect Bisulfite Kit (Qiagen), following the protocol, the reaction mixture containing 1 *μ*g of the DNA, 85 *μ*l of bisulfite mixture and 35 *μ*l of protective DNA solution was prepared. The programme used for conversion with bisulfite was: 95°C 5 minutes; 60°C 25 minutes; 95°C 5 minutes; 60°C 85 minutes; 95°C 5 minutes and 60°C 175minutes. The DNA was then purified by using the kit columns.

### Methylation-specific PCR (MSP)

After bisulfite treatment, 4 *μ*l of the purified DNA was used to perform MSP. The PCRs were performed independently for methylated and unmethylated specific primers under variable conditions for annealing temperatures (55–66°C). All reactions were carried out using the optimised commercial mix Thermo Scientific Maxima Hot Start PCR Mix Master Green (Thermo Scientific). Each reaction mixture contains main reaction mixture, 400 mM sense primer, 400 mM antisense primer, 4 *μ*l of bisulfite-treated DNA and nuclease-free water to make up to a final volume of 25 *μ*l. Primer sequences for the methylated and unmethylated reactions have been reporter by Esteller *et al*. Sequence details for both forward and reverse primers are as follows: forward primer m_MGMT: 5′-TTTCGACGTTCTAGGTTTTCGC-3′; reverse primer m_MGMT: 5′-GCACTCTTCCGAAAACGAAACG-3′; forward primer um_MGMT: 5′-TTTGTGTTTTGATGTTTGTAGGTTTTTGT-3′; reverse primer um_MGMT: 5′-AACTCCACACTCTTCCAAAAACAAAACA-3’. In all reactions, a negative reaction control was added, in which the sample was replaced with water. Negative methylation controls were also added: peripheral blood normal lymphocytes and the LN18 cell line; and positive methylation controls: *in vitro* methylated normal blood lymphocytes *in vitro* with CpG Methyltransferase (M.SssI) (Thermo Scientific), and the LN18 cell line. The amplification was carried out in a Mastercycler Nexus Gradient (Eppendorf) with initial denaturing at 95°C for 5 minutes followed by 35 cycles of denaturing at 95°C for 50 seconds, annealing for 50 seconds at 55–66°C and extension for 50 seconds at 72°C, and then a final extension for 2 minutes at 72°C. The reaction products were analysed by electrophoresis on agarose gels (3%) and stained with a solution of SYBR Safe DNA gel stain (Invitrogen, Life Technologies). The unmethylated or methylated DNA amplicon size was 93bp and 81bp, respectively. The 50bp molecular weight marker (Invitrogen, Life Technologies) was used.

### Reverse transcription from total RNA

SuperScript reverse transcriptase (Invitrogen, Life Technologies) was used to reverse transcription the RNA to cDNA. To do this, the following procedures were adopted: (*i*) 2 *μ*g RNA, incubated with a 2U (1 U/*μ*l) solution of DNase I (Invitrogen, Life Technologies) for 1 hour at 37°C; (*ii*) inactivation of DNase I with 2 *μ*l of 25 mM ethylenediaminetetraacetic acid (EDTA) for 10 minutes at 65°C; (*iii*) incubation with 3 *μ*l of random primers (Invitrogen, Life Technologies) and 1 *μ*l of 10 mM dNTPs (Invitrogen, Life Technologies) for 5 minutes at 56°C, ending with 4°C; (*iv*) incubation with 5 *μ*l of a ribonuclease inhibitor (40U) or RNaseOUT solution (Invitrogen, Life Technologies) for 2 minutes at room temperature; (*v*) incubating with 3 *μ*l of a reverse transcriptase solution (200U) for 10 minutes at room temperature; (*vi*) incubating the mixture for 1 hour at 42°C and then for 15 minutes at 70°C. Control of the reaction was done in parallel without adding reverse transcriptase (called false reverse transcription). The resulting cDNA solution (50 ng/μl) was maintained at –20°C until use.

### Real-time PCR (qPCR) using SYBR Green

The commercially available Fast Start Essential DNA Green Master (Roche) commercial blend was utilised for the reactions. Standard curve was performed to determinate the work concentration. Starting from a concentration of 100 ng/*μ*l cDNA, three different sample dilutions (1/8, 1/16 and 1/32) were performed in duplicate. Each reaction mixture contained: 1X Fast Start Essential DNA Green Master, 400 mM sense primer, 400 nM antisense primer, 2 *μ*l of corresponding cDNA dilution and nuclease free water to complete a final volume of 20 *μ*l. The reactions were performed with the Light Cycler 96 System (Roche). Sequence details for both forward and reverse primers are as follows: forward primer 18S: 5′-CGGCTACCACATCCAAGGAA-3′; reverse primer 18S: 5′-GCTGGAATTACCGCGGCT-3′; forward primer MGMT: 5′-GCTGAATGCCTATTTCCACCA-3′; reverse primer MGMT: 5′-CACAACCTTCAGCAGCTTCCA-3′. Signal detection was performed at the end of the elongation step of the reaction. The conditions of the qPCR were: (*i*) 95°C 10 minutes and (*ii*) 95°C 15 seconds, 60°C 20 seconds, 72°C 20 seconds (45 cycles). The ratio of relative expression of genes to 18S was calculated using the ΔΔCq method. The Cq values were normalised to the values of the 18S housekeeping gene and the results were expressed as –ΔΔCq or Log2 (RQ), thus indicating the change in expression levels between the sample and the calibrator in the form of increments.

### Data collection and statistical analysis

A database was made using the Microsoft Excel 2,000 programme (Microsoft Corporation, USA) with the variables collected without including the identity of the patients. Statistical analysis was performed using SPSS 12.0.0 software. (LEAD Technologies, Chicago, IL, USA). The analysis of the data included the study of the association of variables using the chi-square test and the *t*-Student test. The survival study was performed using the Kaplan–Meier method. The probability value *p* < 0.05 was accepted as significant.

## Results

### Clinical–pathological characteristics

Table 1 presents a summary of the clinical and pathological features of the 50 patients in the study, 40% were male and included 04 of ≤ 21 years old patients (8%). Most cases (58%) came in at least 90% Karnofsky at diagnosis. Histopathological diagnosis was performed under 2007 WHO classification. The temporal localisation was the most frequent (46%) and the median tumour size was 5 (1.5–7 cm). Most patients went to subtotal surgery (60%) and 40% to total surgery, and 74% received radiation, and 60% received chemotherapy, of which 83.33% received temozolomide.

### DNA status in frozen and paraffin tissue samples

**Figure 1.**
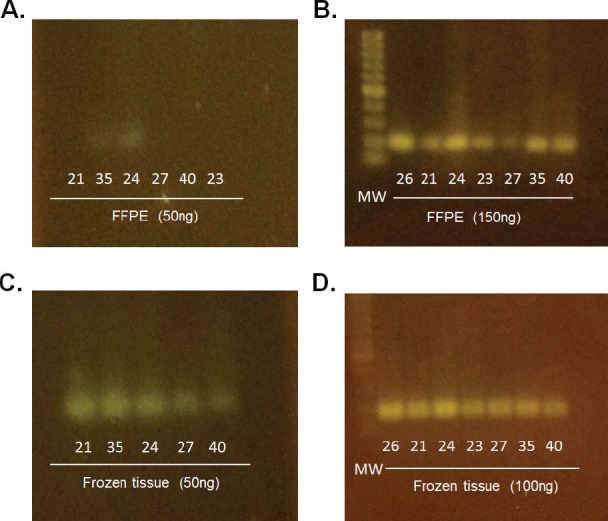
Determination of DNA status in paired paraffin and frozen tissue samples. Visualisation of amplification of the 18S ribosomal gene (18S rRNA) on 3% agarose gel. (A) and (C) Comparison of tumour stored in paraffin-embedded tissue versus frozen in 50ng. (B) and (D) Comparison of tumour stored in paraffin- embedded tissue versus frozen in 150 ng and 100ng, respectively.

In order to evaluate the quality and degradation degree of the DNA obtained from the seven cases in which FFPE and frozen tumour tissue were available, the samples were quantified, analysed by amplification of the 18S ribosomal gene (18S rRNA) and visualised in 3% agarose gel (Figure 1). The results had shown a higher degree of degradation and loss of band intensity in the FFPE samples when compared with the same frozen sample.

### MGMT-promoter methylation status

**Figure 2.**
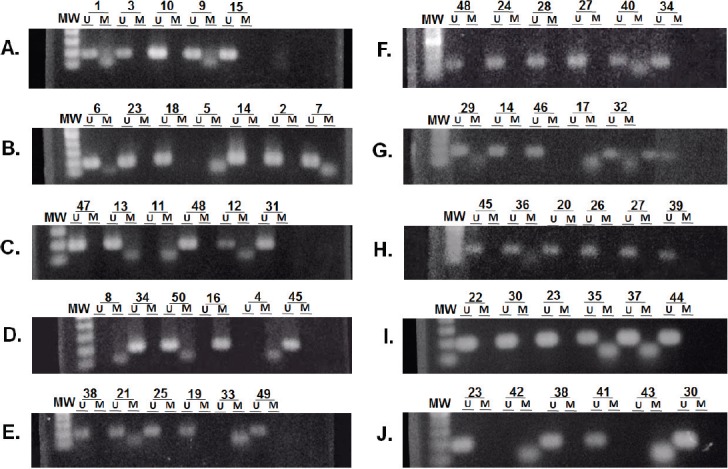
Determination of the methylation status of the *MGMT* promoter. Samples were analysed by MSP. The amplification of the PCR was for unmethylated alleles (U) and methylated alleles (M). The results were visualised on 3% agarose gel. The samples analysed were: (A) 1, 3, 10, 9 and 15. (B) 6, 23, 18, 5, 14, 2 and 7. (C) 47, 13, 11, 48, 12 and 31. (D) 8, 34, 50, 16, 4 and 45. (E) 38, 21, 25, 19, 33 and 49. (F) 48, 24, 28, 27, 40 and 34. (G) 29, 14, 46, 17 and 32. (H) 45, 36, 20, 26, 27 and 39. (I) 22, 30, 23, 35, 37 and 44. (J) 23, 42, 38, 41, 43 and 30.

The methylation status of *MGMT*-promoter was determined in all tumours (*n* = 50) by MSP. Unmethylated (U) alleles were found in 54% (*n* = 27) of the tumours, methylation (M) of both alleles in 20% (*n* = 10) and methylation of only one allele (partial: P) in 26% (*n* = 13) of the tumours (Figure 2). We did not find differences for the methylation status according to age (*p* = 0.164), sex (*p* = 0.647) or tumour size (*p* = 0.876).

The results of methylation status in the HVST experiments were the same as those obtained in INEN, one case with methylated alleles (ID: 4) and another with partial methylation (ID: 7).

The results obtained for paired samples from FFPE and frozen tissue were similar, with partial methylation (ID: 26, 27, 35 and 40, *n* = 4/7) and the absence of methylation in both alleles (ID: 21, 23 and 24; *n* = 3/7). None of the tumour samples had both methylated alleles (*n* = 0/7).

### Association between expression level of the MGMT gene and MGMT-promoter methylation status

**Figure 3.**
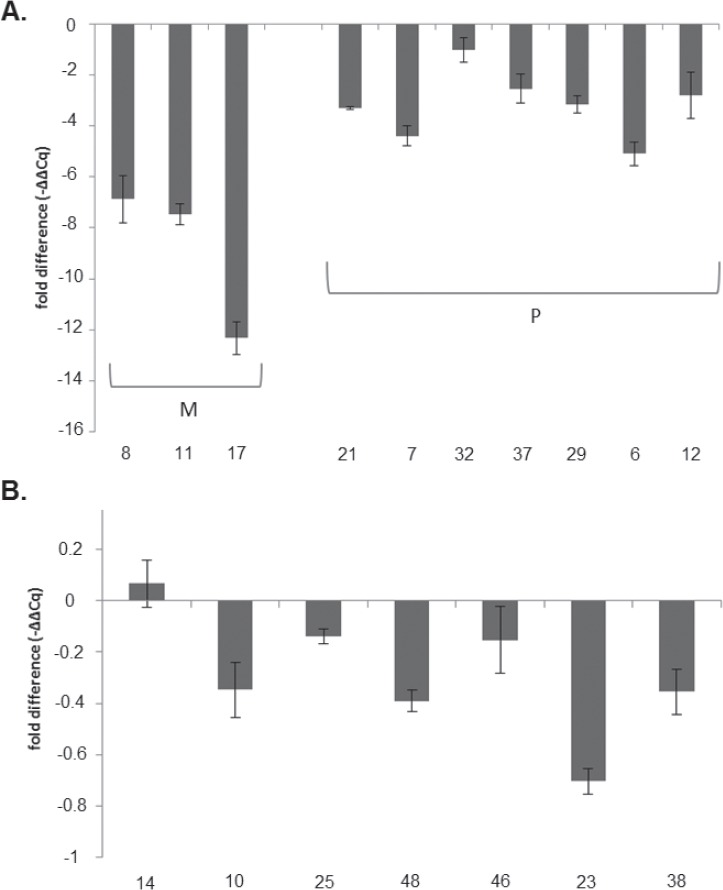
Evaluation of gene expression level of *MGMT* by qPCR. Expression levels of *MGMT* gene were expressed as increments –ΔΔCq and indicated the change in expression levels between tumour samples and the brain tissue calibrator in the form of increments. (A) Expression level of *MGMT* gene for samples with both methylated alleles (M) and with partial methylation (one methylated and one unmethylated allele) (P). (B) Expression level of *MGMT* gene for samples with both unmethylated alleles (U).

The expression level of the *MGMT* gene was determined by real-time PCR (qPCR) in 32.7% of tumours (*n* = 17/50) (Figure 3). The remaining samples could not be evaluated due to poor quality and low concentration at obtained RNA. Expression levels were normalised to the 18s housekeeping gene. Values obtained from samples of healthy brain tissue were used as a calibrator. The results were expressed as -–ΔΔCq, as they evaluated the variation in the expression levels between the samples and the calibrator.

The association analysis between *MGMT* promoter methylation status and *MGMT* gene expression level showed significant differences (*p* = 0.001). The lowest expression levels were found in samples with both methylated alleles (*n* = 3) and had an average of 7.66 ± 0.42 times less than the control. The highest expression levels were observed in the samples with unmethylated alleles (*n* = 7) and had an average of 0.28 ± 0.38 times less than the control. Expression levels in samples with partial methylation (*n* = 7) (one methylated and one unmethylated allele) averaged 3.18 ± 0.40 times less than the control.

### Association between MGMT-promoter methylation status and PFS

**Figure 4.**
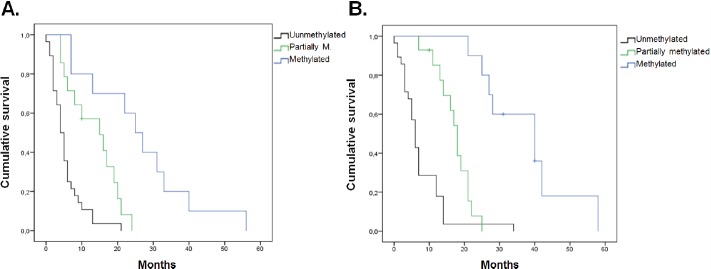
Estimated curve of PFS regarding *MGMT*-promoter methylation status. (A) Estimated curve of OS regarding *MGMT*-promoter methylation status. (B)

The results obtained together with the analysis of clinical and epidemiological information showed a significant association between PFS and all the *MGMT*-promoter methylation status (*p* = 0.002), sex (*p* = 0.029) and degree of resection (*p* = 0.001) (Figure 4). However, no significant association was found between PFS and any age (*p* = 0.527), Karnofsky (*p* = 0.535) and tumour size (*p* = 0.5) variables. According to the Cox regression model, a significant association remained for the presence of methylation (*p* = 0.01) and degree of resection (*p* = 0.004).

### Association between MGMT-promoter methylation status and the global survival

The results showed a significant association between OS and both *MGMT*-promoter methylation status (*p* < 0.001) and degree of resection (*p* < 0.001) (Figure 4). No significant association was found for age (*p* = 0.198), sex (*p* = 0.085), Karnofsky (*p* = 0.318) nor tumour size (*p* = 0.863). According to the Cox regression model, a significant association remained methylation status (*p* < 0.001) and degree of resection (*p* < 0.001).

## Discussion

Establishing and implementing predictive markers of prognosis and response to treatment in clinical practice allow a better patient selection for treatment. The review of the fourth edition of the recently published 2016 WHO Classification has begun to introduce molecular markers including methylation status of *MGMT* promoter; however, most considered information is still limited to traditional anatomo-pathological criteria [13].

Cytosine methylation in different areas of the genome, as in the case of the *MGMT* promoter, has specific patterns associated with race and environmental factors such as diet quality [14, 15] and its effect on the behaviour of the neoplasia may differ according to race [16]. Some studies found that glioblastoma has a higher prevalence in non-Hispanics than in Hispanic population [17, 18]. The present study describes, for the first time to our knowledge, a rate of *MGMT*-promoter methylation status in Peruvian cases of glioblastoma (46%) that is in the range described for Caucasian race (36–50%) [19–24].

To evaluate the quality of our samples, we compared the quality and degree of degradation of DNA obtained from FFPE and from frozen samples. The evaluation revealed a higher degree of degradation and loss of band intensity in the FFPE samples. Similarly, Sanchez-Navarro *et al* evaluated the quantitative data correlation between fresh and FFPE tissue samples. They found that the RNA extracted from FFPE samples had shown a variation in the basal values of Cq that can be explained by extensive degradation. However, adequate normalisation may compensate the effects of RNA degradation on the measurement of gene expression. They also found that the correlation among normalised expression values is better for genes of moderate-high expression [25, 26].

The analysis of two paraffin samples was repeated in other institution and confirmed our results. A pilot study comparing protocols for determination of the *MGMT* promoter status in 23 centres in Germany, Austria and the Netherlands found a good concordance rate in the group with methylation of both alleles, whereas differed according to the laboratory in the group with partial methylation [27]. The conference on the European consensus for the measurement of external quality in molecular pathology carried out in 2012 recommended the implementation of certification programs that include standardised inter-laboratory comparison at international level [28].

We evaluated the relationship between promoter methylation status and *MGMT* gene expression in 17 cases, and found an adequate correlation for the group of cases with unmethylated alleles and for the methylated allele group (*p* = 0.001). Similarly, Bady *et al* compared the promoter status and *MGMT* gene expression in the same samples and found an adequate correlation between both values and between these values and survival [29]. MSP only indicates whether or not the cytosine residues are methylated or not in the *MGMT* promoter, but not, if there is loss of expression of the *MGMT* gene. Therefore, direct analysis of the expression of MGMT allows corroborating that there are no alternative routes or modifications [24].

Testing methylation of *MGMT* promoter in glioblastoma has been implemented in centres of reference in cancer worldwide and our results confirm the prognostic value of the methylation status in our population [5, 19, 21, 22, 24]. Thus, we can state that the methodology used in our study to detect epigenetic changes in tumours can be reliably implemented in the routine practice of glioblastoma patients in our cancer institute, and our analysis could be replicated and implemented in other Latin American centres. Incorporation of this technique will allow to select those cases with greater benefit to alkylating chemotherapy and to classify the Latin American population under molecular classification.

The strength of our study is that it used the PCR methodology which is the test of choice for evaluating methylation because of its simplicity and easy access which allows its implementation in Latin American countries [19–24]. Some recent studies have suggested that a quantitative analysis of the *MGMT*-promoter methylation through sequencing technology correlates better with survival, however, it is a more expensive methodology [30].

A weakness of our series is the small size sample that needs to be confirmed in other Latin American series.

## Conclusion

The rate of *MGMT*-promoter methylation in Peruvian glioblastoma cases is similar to that described in other races and PCR for its detection in FFPE of glioblastoma samples is an accurate methodology to implement in Latin American countries.

## Conflict of interests

The authors declare that they have no conflict of interests.

## Institutional review

The conduct of this survey was approved by The Institutional Review Board of INEN (#051-2016-CRP-DI-DICON/INEN). Since the study was based on a secondary source and there was no contact with the patients, no informed consent was applied; however, the identity and personal data of patients’ medical records were protected at all times.

## Authors’ contributions

Belmar-Lopez C, Castaneda CA, Castillo M, Garcia-Corrochano P and Orrego E contributed to the conception and design of the study and performed data analysis and interpretation; Casavilca S, Barbara M and Claudio F performed the data acquisition, as well as providing administrative, technical, and material support; all authors drafted the article and made critical revisions related to the intellectual content of the manuscript, and approved the final version of the article to be published.

## Figures and Tables

**Table 1.** Clinical and pathological characteristics.

Features	*n*	%
Patients	50	100
Age (year)		
Mean (range)	51 (8–74)	
> 60	17	34
Gender		
Male	20	40
Female	30	60
Karnofsky scale		
70	10	20
80	11	22
> 90	29	58
Location		
Temporal	23	46
Frontal	18	36
Occipital	9	18
Parietal	9	18
Symptomas		
Seizures	15	30
Focalisation	30	60
Larger tumour diameter (cm)		
Average/Range	5 (1.5–7.0)	
< 5	19	38
> 5	31	62
Histological diagnosis		
Glioblastoma	38	76
G. Comp. Oligodendroglial	3	6
G. Giant Cells	3	6
G. Small Cells	2	4
G. PNET	2	4
Gliosarcoma	2	4
*MGMT*-promoter methylation status		
Unmethylated alleles	27	54
Partially methylated alleles	13	26
Methylated alleles	10	20
Surgery		
Subtotal surgery	30	60
Total surgery	20	40
Adjuvant treatment		
Radiotherapy	37	74
Chemotherapy	30	60
Temozolomida	25	83.33
Carmustine	2	13.33
Bevacizumab	2	4
